# Genome-Wide Analyses of the NAC Transcription Factor Gene Family in Pepper (*Capsicum annuum* L.): Chromosome Location, Phylogeny, Structure, Expression Patterns, *Cis*-Elements in the Promoter, and Interaction Network

**DOI:** 10.3390/ijms19041028

**Published:** 2018-03-29

**Authors:** Weiping Diao, John C. Snyder, Shubin Wang, Jinbing Liu, Baogui Pan, Guangjun Guo, Wei Ge, Mohammad Hasan Salman Ali Dawood

**Affiliations:** 1 Jiangsu Key Laboratory for Horticultural Crop Genetic Improvement, Institute of Vegetable Crops, Jiangsu Academy of Agricultural Sciences, Nanjing 210014, China; diaowp@jaas.ac.cn (W.D.); liujinbing@jaas.ac.cn (J.L.); pantix@jaas.ac.cn (B.P.); guoguangjun@jaas.ac.cn (G.G.); gewei@jaas.ac.cn (W.G.); 2 College of Agricultural, Food and Environment, University of Kentucky, Lexington, KY 40546, USA; snyder@email.uky.edu (J.C.S.); mohammadh.salman@uky.edu (M.H.S.A.D.)

**Keywords:** pepper, transcription factor, NAC family, phylogenetic, gene expression, interaction network

## Abstract

The NAM, ATAF1/2, and CUC2 (NAC) transcription factors form a large plant-specific gene family, which is involved in the regulation of tissue development in response to biotic and abiotic stress. To date, there have been no comprehensive studies investigating chromosomal location, gene structure, gene phylogeny, conserved motifs, or gene expression of NAC in pepper (*Capsicum annuum* L.). The recent release of the complete genome sequence of pepper allowed us to perform a genome-wide investigation of *Capsicum annuum* L. NAC (*CaNAC*) proteins. In the present study, a comprehensive analysis of the *CaNAC* gene family in pepper was performed, and a total of 104 *CaNAC* genes were identified. Genome mapping analysis revealed that *CaNAC* genes were enriched on four chromosomes (chromosomes 1, 2, 3, and 6). In addition, phylogenetic analysis of the NAC domains from pepper, potato, *Arabidopsis*, and rice showed that *CaNAC* genes could be clustered into three groups (I, II, and III). Group III, which contained 24 *CaNAC* genes, was exclusive to the *Solanaceae* plant family. Gene structure and protein motif analyses showed that these genes were relatively conserved within each subgroup. The number of introns in *CaNAC* genes varied from 0 to 8, with 83 (78.9%) of *CaNAC* genes containing two or less introns. Promoter analysis confirmed that *CaNAC* genes are involved in pepper growth, development, and biotic or abiotic stress responses. Further, the expression of 22 selected *CaNAC* genes in response to seven different biotic and abiotic stresses [salt, heat shock, drought, *Phytophthora capsici*, abscisic acid, salicylic acid (SA), and methyl jasmonate (MeJA)] was evaluated by quantitative RT-PCR to determine their stress-related expression patterns. Several putative stress-responsive *CaNAC* genes, including *CaNAC*72 and *CaNAC*27, which are orthologs of the known stress-responsive *Arabidopsis* gene *ANAC*055 and potato gene *StNAC*30, respectively, were highly regulated by treatment with different types of stress. Our results also showed that *CaNAC*36 plays an important role in the interaction network, interacting with 48 genes. Most of these genes are in the mitogen-activated protein kinase (MAPK) family. Taken together, our results provide a platform for further studies to identify the biological functions of *CaNAC* genes.

## 1. Introduction

Transcriptional regulation of gene expression controls many important cellular processes in plants, such as environmental stress responses, signal transduction, and cellular morphogenesis. Transcription factors (TFs) regulate gene expression by binding to specific *cis-*acting promoter elements, resulting in the activation or repression of the transcriptional rates of target genes, thereby facilitating the evolution and adaption of complex developmental systems in plant responses to environmental stress. Many plant-specific transcription factors, such as NAC, WRKY, AP2/EREBP, and MYB proteins have been identified and shown to play an important role in defense responses to stress [1,2].

NAC proteins are encoded by one of the largest plant-specific transcription factor gene families and contain both highly conserved N-terminal DNA binding domains and variable C-terminal domains. The conserved N-terminal DNA binding domain consists of five subdomains, A through E. On the basis of the structure of the domains and subdomains, NACs can be classified into two main groups (I and II), which can be further divided into 14 and 4 subgroups according to their tree topology in *Arabidopsis* and rice [3].

Since NAC transcription factors were originally identified as petunia NAM [4] and *Arabidopsis* CUC2 [5], many NAC proteins have been identified from virtually all classes of plants. As an increasing number of whole plant genomes are sequenced, genome-wide identification and functional analysis of NAC transcription factors have been accomplished in species such as *Arabidopsis thaliana* [3], rice [6], soybean [7], apple [8], foxtail miller [9], potato [10], Chinese cabbage [11], cassava [12], maize [13], melon [14], and wheat [15].

Many studies of NAC identification and functional analysis have shown that NAC proteins play significant roles in signaling and regulation of gene expression in response to biotic and abiotic stresses. Studies have shown that in *Arabidopsis*, the NAC transcription factor ANAC096 cooperates with the bZIP-type TF abscisic acid responsive element (ABRE) binding factor and the ABRE binding protein (ABF/AREB) to help plants survive under dehydration and osmotic stress conditions [16]. The overexpression of *ANAC*055 significantly improved drought resistance in transgenic plants [17]. In addition, the transcription of *SNAC*3 is induced by drought, high temperature, salinity stress, and ABA treatment in rice. Overexpression of *SNAC*3 resulted in enhanced tolerance to high temperatures, drought, and oxidative stress, whereas suppression of *SNAC*3 by RNAi resulted in increased sensitivity to these stresses [18]. In apple, a total of 180 *NAC* genes were identified, and 17 of 29 selected *MdNAC* genes were differentially expressed in response to at least one abiotic stress (low temperature, drought, high salinity, or exogenous ABA) [8]. In cassava, the expression profiles of *MeNAC* genes under various conditions (osmotic, salt, cold, ABA, and H_2_O_2_) suggest that different *MeNAC* genes may participate in different signaling or stress responses, and most of the cassava *NAC* genes can be significantly induced by multiple stressors, such as ABA and H_2_O_2_ treatment [12]. In maize, three genes, *ZmNAC*18, *ZmNAC*51, and *ZmNAC*145 were upregulated in the drought-tolerant genotype and downregulated in the susceptible genotype. These three genes are closely linked to *SNAC*1 and *OsNAC*6 in rice, which have been reported as drought responsive, and act as transcriptional activators/regulators of genes that encode proteins involved in the regulation of stress responses and the production of osmolytes [13]. In potato, 48 *StNAC* genes were expressed under different abiotic stress treatments (salt, mannitol, and heat), and 44 *StNAC* genes were expressed under biotic stress conditions with *P. infestans* inoculum (Pi isolate US8:Pi02-007), acibenzolar-s-methyl (BTH) and DL-β-amino-n-butyric acid (BABA) [10]. Meanwhile, the NAC transcription factor *SISRN*1 positively regulates the defense response against *Botrytis cinerea* and *Pseudomonas syringae* pv. tomato DC3000, but negatively regulates the oxidative and drought stress response in tomato [19].

Compared to potato and tomato, the few *NAC* genes identified were shown to display tissue-specific, inducible expression patterns in *Capsicum*. The first *NAC* gene, i.e., *CaNAC*1, was cloned by reverse RNA gel blot analysis. It was shown that the expression of the *CaNAC*1 gene is rapidly and specifically induced during incompatible interactions between pepper and bacterial or viral pathogens. Additionally, *CaNAC*1 was strongly induced by exogenously applied SA and ethephon (ET), whereas MeJA only had a transient effect [20]. The full-length cDNA of *CaNAC*72, a gene encoded by NAC transcription factors, was isolated from the normalized cDNA library. It was shown that this gene shares 70% amino acid sequence identity with the *AtNAC*072 gene of *Arabidopsis*. The expression of *CaNAC*72 was induced by hormones (SA, MeJA, ET, and ABA), dehydration, high salinity, cold, heat, and *Ralstonia solanacearum* inoculation [21]. The transcription expression of *CaNAC*2 was strongly induced by abiotic stress treatments, such as cold, salt, and ABA, but was inhibited by osmotic stress and SA treatment. Viral silencing of *CaNAC*2 in pepper seedlings resulted in increased susceptibility to cold stress and delayed salt-induced leaf chlorophyll degradation [22]. Although 73 and 45 putative NAC sequences were identified from a wild Mexican pepper accession “Chiltepin” and a Chinese inbred derivative “Zunla-1”, respectively [23], detailed analyses including gene classification, chromosome distribution, gene duplication, gene phylogeny, gene structure, conserved motif composition, and gene expression under various abiotic and biotic stress conditions was lacking.

Pepper (*Capsicum annuum* L.) is the second most widely cultivated *Solanaceous* vegetable worldwide, after tomato. However, when in the reproductive stage, pepper is sensitive to biotic and abiotic stresses such as pathogens, drought, cold, and high temperatures. The NAC transcription factors are one of the largest families of transcriptional regulators in plants, and members of the *NAC* gene family are thought to play important roles in the regulation of transcriptional reprogramming associated with plant abiotic and biotic stress responses [24]. Thus, it is necessary to identify NAC transcription factors and determine the molecular mechanisms involved in the multiple regulatory biological processes in pepper. Drafts of the *C. annuum* L. genome sequence were reported recently [25,26]. In the present study, we searched two pepper genome sequences to identify the *NAC* genes (*CaNAC*). Detailed analyses were then conducted, including gene classification, chromosome distribution, gene structure, gene duplication, gene phylogeny, conserved motif composition, and *cis*-acting elements in the promoter. Further, we analyzed the expression of the identified 22 *CaNAC* genes under abiotic or biotic stress conditions (salt, heat shock, drought, *Phytophthora capsici*, ABA, SA, and MeJA), and predicted the protein–protein interaction network of *CaNAC*s. These results will provide a platform for the future identification of the biological functions of *NAC* genes in pepper.

## 2. Results

### 2.1. Identification of NAC Family Members in the Capsicum Annuum Genome

A comprehensive analysis was performed to identify *NAC* genes in the pepper genome sequences downloaded from PGP (Available online: http://peppergenome.snu.ac.kr) and PGD (Available online: http://peppersequence.genomics.cn). A total of 104 non-redundant putative *NAC* genes were identified using two pepper genome databases and named *CaNAC*1 to *CaNAC*104. The *NAC* gene family in the pepper is comprised of >100 genes, like those reported for *Arabidopsis*, rice, potato, apple, and soybean. The annotation IDs of each *CaNAC* in two pepper genome databases (PGP and PGD) are given in Table 1. Ten genes (*CaNAC*8, *CaNAC*9, *CaNAC*42, *CaNAC*44, *CaNAC*47, *CaNAC*48, *CaNAC*58, *CaNAC*64, *CaNAC*68, *CaNAC*89) have only one annotation ID because of the specificity of their gene sequences. It is interesting to note that these ten genes only exist in the PGD database. The nucleotide and protein sequences of *CaNAC* gene family were also (File S1).

Typically, the NAC proteins have an N-terminal NAC domain and a C-terminal activation domain. The NAC domain can be further divided into five subdomains, A to E [3]. All of the putative 104 *NAC* genes were analyzed to confirm the presence of the NAC domain, and 97 *CaNAC* genes, containing a complete N-terminal NAC domain, were identified; another seven genes did not have complete domains. Thereinto, *CaNAC*48 has only a D subdomain, *CaNAC*4 and *CaNAC*47 have D and E subdomains, *CaNAC*64 and *CaNAC*82 have C and D subdomains, and *CaNAC*31 and *CaNAC*68 have C, D, and E subdomains. Similar phenomena were observed in the potato, where 13 *StNAC*s lack conserved A and/or B subdomains, and four *StNAC*s do not contain conserved C and/or D subdomains [10].

The 104 identified CaNAC proteins varied greatly in length, from 100 amino acids (aa, AA) (*CaNAC*2) to 719 aa (*CaNAC*96); the average NAC protein length was 331.9 aa. The relative WT (molecular weight) varied from 11.80 kDa (*CaNAC*2) to 79.51 kDa (*CaNAC*96), and the PI (isoelectric point) ranged from 4.08 (*CaNAC*16) to 10.32 (*CaNAC*82). The detailed information about *CaNAC* proteins, including gene loci accession number in PGP or PGD, chromosome location, gene classification, introns, PI, AA, WT, and *Arabidopsis thaliana* orthologous genes or orthologs, are listed in Table 1.

### 2.2. Chromosomal Distribution and Duplication of CaNAC Genes

**Figure 1:**
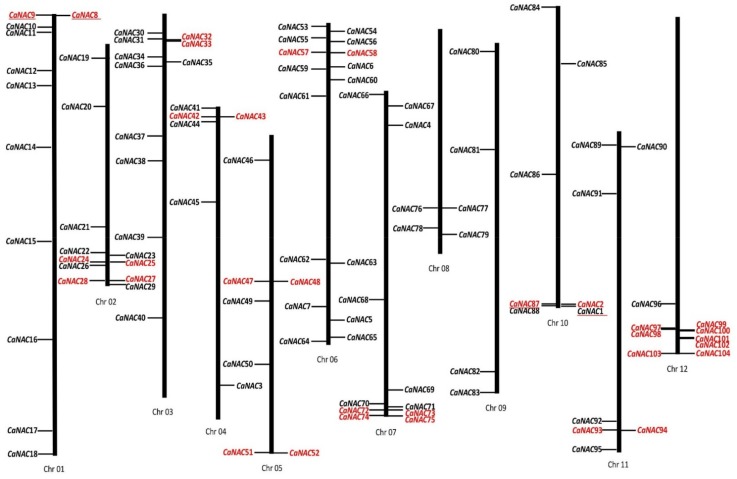
Mapping of the *NAC* gene family on *Capsicum annuum* L. chromosomes. The size of each chromosome is indicated by its relative length. Tandemly duplicated genes are indicated in red. Segmental duplicated genes are underlined.

*CaNAC*s were widely distributed within the *Capsicum annuum* reference genome, with all 104 *CaNAC* genes mapped to 12 chromosomes (Figure 1). Chromosomes 1, 2, 3, 6, and 7 contained relatively more *CaNAC* genes, with 11, 11, 11, 16, and 11 genes, respectively, compared to chromosomes 8 and 9 which each possessed only four of the genes. Interestingly, *CaNAC* genes were enriched on chromosomes 2 and 6. The sequenced size of chromosome 2 is 156.37 M, and it contains 11 (10.6%) of the 104 *CaNAC* genes; the sequenced size of chromosome 6 is 209.35 M, and it contains 16 (15.4%) *CaNAC* genes. Further, chromosomes 2 and 6 account for only 4.87 and 6.53% of the assembled pepper genome (3.13 G). Similar to pepper, chromosome 2 in potato also contains the largest number of *StNAC* genes, comprising 14 members (~13%), whereas chromosome 9 in potato contains only three members (~3%) [10]. These data suggest that in *Solanaceae*, *NAC* genes are enriched on chromosome 2, but not chromosome 9; however, the distribution of *NAC* genes in other *Solanaceae* family plants, such as tomato and eggplant, has yet to be determined.

Further, we determined the tandem and segmental duplications of *CaNAC* genes along the 12 pepper chromosomes. As shown in Figure 1, 16 *CaNAC* gene clusters (genes labeled in red) containing 32 tandemly duplicated genes were identified on 10 chromosomes, except for chromosomes 8 and 9. Interestingly, we identified eight tandemly duplicated genes within four gene clusters on chromosome 12. The sequences of the clustered members are highly alike, suggesting the use of gene duplication in the expansion of the *CaNAC* family. However, the number of segmental duplicated genes was lower compared to the number of tandem duplicated genes, containing only two pairs and four genes (*CaNAC*1 to *CaNAC*87 and *CaNAC*8 to *CaNAC*9) (Figure 1).

### 2.3. Classification of CaNACs

**Figure 2:**
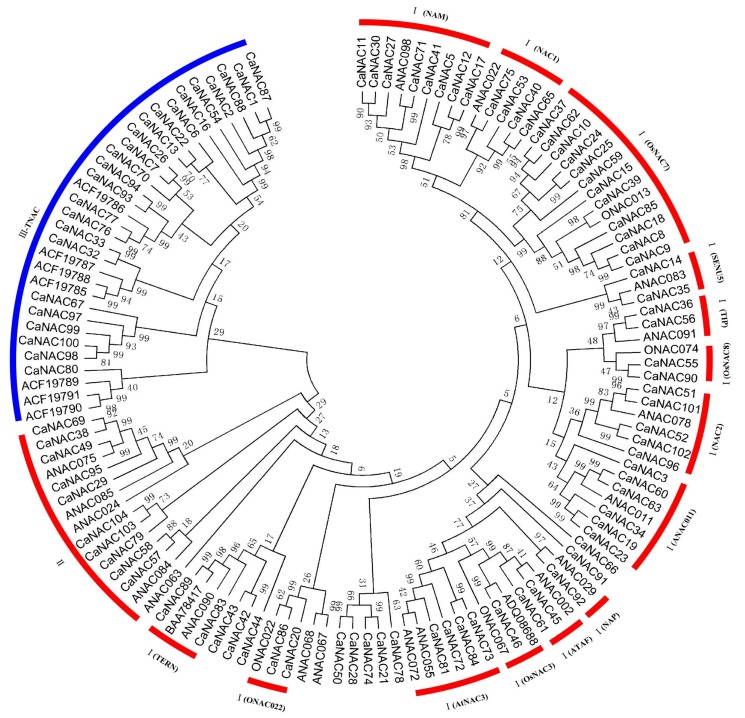
Unrooted phylogenetic tree representing the relationships among the NAC domains of pepper, potato, rice, and *Arabidopsis*. The amino acid sequences of all NAC domains were aligned with Clustal W (University College Dublin, Belfield, Dublin, Ireland), and the phylogenetics tree was constructed using the neighbor-joining method in MEGA 6.06 (Tokyo Metropolitan University, Hachioji, Tokyo, Japan). The red arcs indicate different groups or subgroups of NAC domains.

The most prominent structural feature of NAC proteins is the conserved NAC domain, which is divided into five subdomains at its N-terminal region. The phylogenetic relationship of the 97 *CaNAC* proteins and 31 known representative NAC proteins belonging to different subgroups from *Arabidopsis*, rice, and potato was examined by multiple sequence alignment of their NAC domain sequences. Because of short sequences or the lack of complete subdomains, the other seven *NAC* genes cannot be used for phylogenetic tree construction. Based on the *ANAC*, *ONAC*, and *TNAC* classification [10] and NAC domain alignments of *CaNAC*s, 97 *CaNAC*s were classified into three main groups (Groups I, II, and III) which contained 63, 10, and 24 pepper NAC members, respectively (Figure 2). Further, the *CaNAC* domains in each main group could be divided into several subgroups based on similarities in NAC domain structures.

Fourteen subgroups in Group I, and four subgroups in Group II have been identified in *Arabidopsis* and rice [3]. In this study, 14 subgroups were also identified in the Group I *NAC* gene family in pepper. The number of *CaNAC* genes in each subgroup varied greatly, as shown in Figure 2. Subgroup I (*OsNAC*3) and I (NAP) possessed only one gene, respectively. Subgroup I (*OsNAC*7) and I (NAM) contained relatively more *CaNAC* genes, with 12 and 8 genes, respectively. Further, we identified four subgroups in the pepper Group II *NAC* gene family, which is similar to *Arabidopsis* and rice, where the Group II *NAC* gene family contains the four subgroups (*ANAC*001, *ONAC*003, *ONAC*001, and *ANAC*063). Interestingly, we found that Group III contains 24 *CaNAC* genes, but no *Arabidopsis* and rice NAC. The 24 *CaNAC* genes are included into the *TNAC* subfamily, which is thought to be exclusive to the *Solanaceae* family [10,27]. Similar phenomena were observed in tobacco and potato. Here, we confirmed the existence of a *Solanaceae*-specific *NAC* subfamily*.*

**Figure 3:**
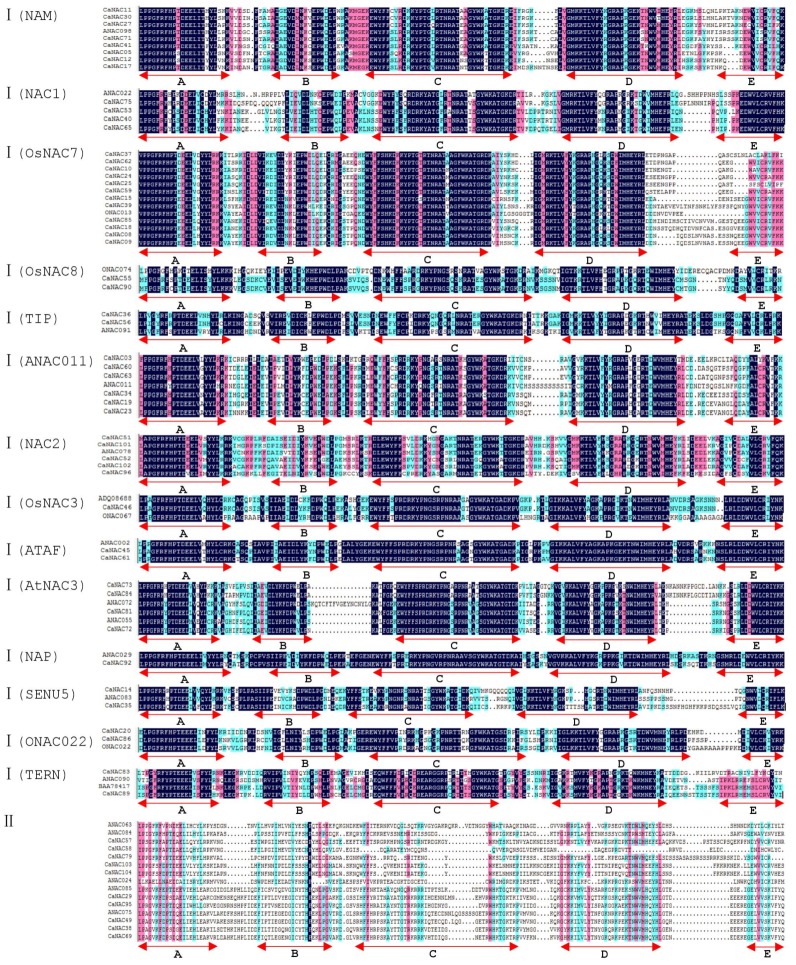
Sequence alignment of two groups of *CaNAC* domains amino acid sequences. Subdomains A to E are shown by arrows above the sequences. Amino acids in the consensus sequences that are common to all groups are shown in black (=100%). Amino acids in the consensus sequences that are common to 20–25 subgroups (≥75%) are shown in blue, and those common to 13–19 (≥50%) are shown in green.

Further, we determined the characteristic features of each group and subgroup using the sequence alignments of the quantified sequences of NAC domains. As shown in Figure 3, subdomains A, C, and D were tightly conserved, whereas subdomains B and E were relatively divergent, except in subgroups 4, 5, and 6 of Group I. The sequences of subdomains B and E in subgroups 4, 5, and 6 were almost identical, akin to observations in *Arabidopsis* [3]. In this regard, pepper is similar to *Arabidopsis* and rice. Compared with Group I, the sequences of NAC in Group II from *Arabidopsis* or rice have low similarity to *CaNAC* in pepper, except for subgroup *ANAC*075 (Figure 3).

### 2.4. Gene Structure and Motif Composition Analysis of CaNAC Proteins

**Figure 4:**
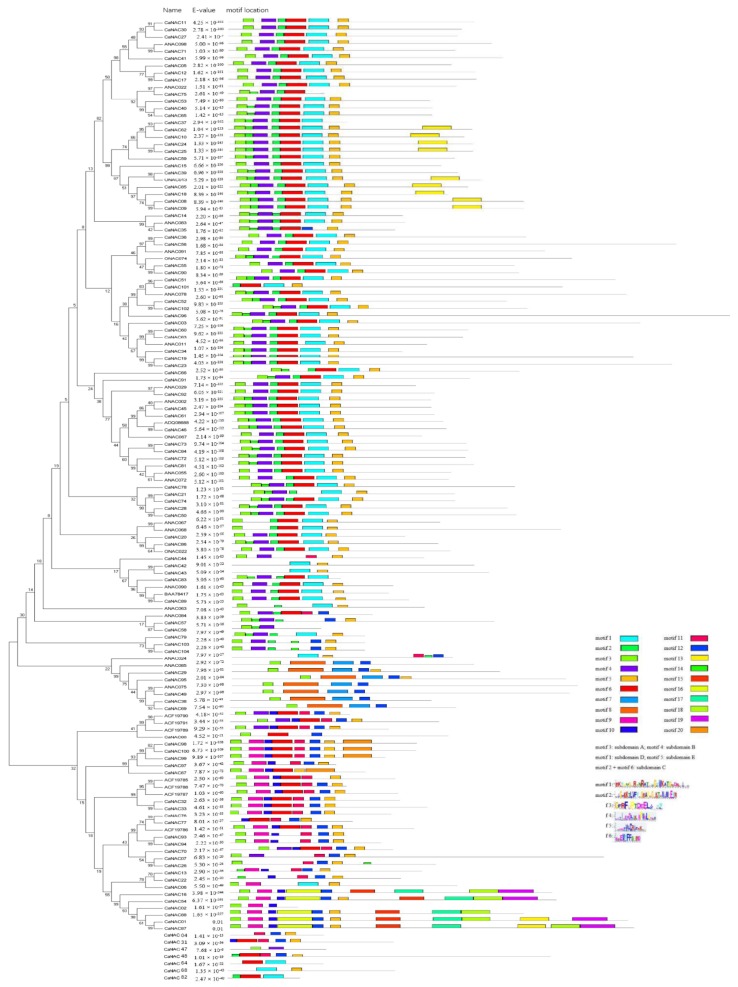
Alignment of multiple *CaNAC* and selected *ONAC*, *ANAC* domain amino acid sequences, schematic diagram of amino acid motifs of *CaNAC* and *ONAC*, *ANAC* protein groups or subgroups. Motif analysis was performed using Meme 4.12.0 online software (Available online: http://meme-suite.org/tools/meme) as described in the methods. The NAC proteins are listed on the left. The different-colored boxes represent different motifs and their position in each NAC sequence. The sequences of key motifs (motif 1, motif 2, motif 3, motif 4, motif 5, and motif 6) are shown on the bottom right of the figure. A detailed motif introduction for all *CaNAC* protein is shown in File S2.

To better understand the similarity and diversity of *CaNAC* proteins, the intron/exon distribution of *CaNAC* was examined, and the conserved motifs of NAC family proteins in pepper, potato, *Arabidopsis*, and rice were also investigated using MEME version 4.12.0 online software (Available online: http://meme-suite.org/tools/meme). Gene structure analysis indicated that the number of introns of *CaNAC* ranged from 0 to 8 (Table 1). In particular, 26 and 39 *CaNAC* genes contained zero and two introns, respectively. Eighty-three (78.9%) *CaNAC* genes contained two or less introns, which is similar to cassava and banana, indicating low structural diversity of *CaNAC* genes. In addition, the *CaNAC*73 gene possessed eight introns. As shown in Figure 4 and File S2, a total of 20 distinct motifs were identified, and a schematic overview of the identified motifs is provided. Interestingly, most of the conserved motifs located in the N-terminal of NAC proteins are highly conserved for DNA-binding, and a similar motif composition is shared by the same subgroups, indicating functional similarities among members of the same subgroups.

*CaNAC*1, *CaNAC*54, and *CaNAC*87 contained relatively more motifs, (11, 11, and 10 motifs, respectively), compared to *CaNAC*42, *CaNAC*43, and *CaNAC*47, which contained only 2 motifs each. Among the 20 motifs in Group I, motif 3, motif 4, motif 1, and motif 5 comprised the subdomains A, B, D, and E, respectively, and motifs 2 and 6 together comprised subdomain C. The number and kind of motifs were almost identical in Group I. However, motif 13 was unique in subgroup I (*OsNAC*7). Compared to Group I, the number and kind of motifs were diverse in Group II and III. Motif 3 existed in all *CaNAC* genes, where motifs 2 and 4 were only represented in subgroup II (*ANAC*084). Further, motifs 1 and 6 were not found in Group II. Two unique motifs (motifs 7 and 8) were identified in subgroup II (*ONAC*003), and motifs 8, 7, and 12 comprised the subdomain B, C, and D, respectively. Motif 12 was identified in most *CaNAC* genes in Group II and III. In Group III, motifs 3, 5, 9, 10, and 11 were found in most *CaNAC* genes. Motif 9 comprised subdomain B, motifs 6 and 10 together comprised subdomain C, and motifs 11 and 12 together comprised subdomain D. Interestingly, we found that five motifs (15, 16, 17, 18, and 19) were always represented in five genes and that subdomain C in these five *CaNAC* genes was comprised of motif 16.

Seven *NAC* genes, *CaNAC*4, *CaNAC*31, *CaNAC*47, *CaNAC*48, *CaNAC*64, *CaNAC*68, and *CaNAC*82 cannot be divided into any subgroups because they cannot be used for phylogenetic tree construction as a consequence of their shortened sequences or lack of complete subdomains. However, as displayed schematically in Figure 4, *CaNAC*64, *CaNAC*68, and *CaNAC*82 can be divided into Group I because they contain motif 2. Overall, the number and kind of motifs in Group I, Group II, and Group III are quite different, indicating that the functions of these three main groups have been diversified.

### 2.5. Expression Profiling of CaNAC Genes under Biotic and Abiotic Stresses

We analyzed the expression patterns of 22 *CaNAC*s belonging to the different subgroups under normal growth conditions and various biotic and abiotic stress conditions using real-time quantitative RT-PCR.

#### 2.5.1. Expression of Patterns of *CaNAC* Genes under Salt, Heat, and Drought Treatment

**Figure 5:**
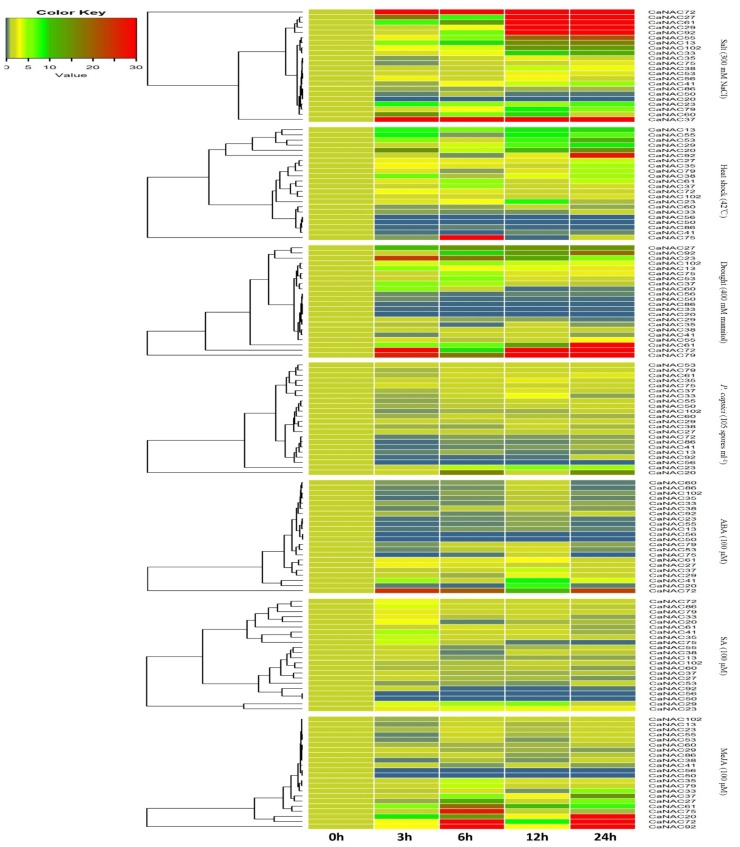
Heat map showing *CaNAC* genes expression pattern in pepper under seven different types of stress. The leaves or roots of the seedlings (six true leaves) were used to test the changes of *CaNAC* gene expression levels using real-time PCR at different timepoints (0, 3, 6, 12 and 24 h) with salt, heat shock, abscisic acid (ABA), salicylic acid (SA), methyl jasmonate (MeJA), drought, and *Phytophthora capsici* treatment, respectively. *Actin*1 was used as an internal control. qRT-PCR data are shown relative to 0 h. The relative expression levels were calculated using the 2(−∆*C*t) method. The heat map was created using R language.

Under salt treatment, only the expression of one gene, *CaNAC*20, was not detectable in the leaves of the treatment groups. However, the expression of 10 genes was significantly upregulated in response to stress treatment, increasing on average by more than 10-fold after treatment (Figure 5, genes labeled in red or dark green). Of note, *CaNAC*72 was induced by more than 600-fold after 12 h of salt treatment. As shown in Figure 5, the expression of *CaNAC*50 and *CaNAC*86 were downregulated, while the expression of *CaNAC*50 significantly decreased by 0.2-fold after 24 h of treatment.

Under heat shock treatment, as shown in Figure 5, *CaNAC*50 and *CaNAC*56 were not expressed in the leaves of the treated plants. The expression of four genes (*CaNAC*13, *CaNAC*20, *CaNAC*29, and *CaNAC*53) was significantly upregulated following stress treatment. The expression of *CaNAC*27, *CaNAC*35, *CaNAC*37, *CaNAC*61, *CaNAC*72, and *CaNAC*102 was also upregulated. Meanwhile, the expression value of seven genes (*CaNAC*13, *CaNAC*20, *CaNAC*27, *CaNAC*29, *CaNAC*35, *CaNAC*53, and *CaNA*102) peaked after 24 h of treatment. In addition, the expression of *CaNAC*41 and *CaNAC*86 was significantly decreased by 0.02- and 0.06-fold after 12 and 24 h of heat shock treatment, respectively.

As shown in Figure 5, the expression of *CaNAC*20 was not detectable in the roots after drought treatment. Six genes (*CaNAC*23, *CaNAC*27, *CaNAC*61, *CaNAC*72, *CaNAC*79, and *CaNAC*92) were expressed with relatively higher intensities and showed significant upregulation; *CaNAC*72 was induced by more than 77-fold after 3 h, and *CaNAC*79 was induced by more than 110-fold after 12 h of drought treatment. Conversely, the expression of six genes (*CaNAC*29, *CaNAC*33, *CaNAC*35, *CaNAC*50, *CaNAC*56, and *CaNAC*86) was downregulated, significantly decreasing by 0.65-, 0.04-, 0.17-, 0.13-, 0.2-, and 0.06-fold after 3 h of drought treatment, respectively.

#### 2.5.2. Expression Patterns of *CaNAC* Genes under *P. capsici* Inoculation

As shown in Figure 5, *CaNAC*56 expression was not detectable in the roots of plants in the *P. capsici* inoculation treatment groups. The expression of five genes (*CaNAC*20, *CaNAC*23, *CaNAC*35, *CaNAC*53, and *CaNA*75) was upregulated, and six genes (*CaNAC*13, *CaNAC*41, *CaNAC*72, *CaNAC*86, and *CaNA*102) were downregulated. The expression value of seven genes (*CaNAC*23, *CaNAC*29, *CaNAC*33, *CaNAC*35, *CaNAC*37, *CaNAC*38, and *CaNA*53) peaked after 12 h of treatment. Among these genes, *CaNAC*23 was induced by more than 5.0-fold after 12 h of *P. capsici* inoculation treatment. Further, the expression value of nine genes (*CaNAC*13, *CaNAC*41, *CaNAC*50, *CaNAC*55, *CaNAC*60, *CaNAC*61, *CaNAC*72, *CaNAC*79, *CaNAC*86, and *CaNA*102) peaked after 3 h of treatment.

#### 2.5.3. Expression Patterns of *CaNAC* Genes under Hormone Treatment

Under ABA treatment, the expression of five genes (*CaNAC*27, *CaNAC*37, *CaNAC*41, *CaNAC*61, and *CaNAC*72) was upregulated, and seven genes (*CaNAC*13, *CaNAC*23, *CaNAC*33, *CaNAC*35, *CaNAC*38, *CaNAC*55, and *CaNAC*102) were downregulated. The expression value of two genes (*CaNAC*27 and *CaNAC*72) and three genes (*CaNAC*37, *CaNAC*41, and *CaNAC*61) peaked after 6 h and 12 h of treatment, respectively. In addition, the expression of five other genes (*CaNAC*20, *CaNAC*53, *CaNAC*60, *CaNAC*75, and *CaNAC*86) was downregulated, except after 12 h of treatment. As shown in Figure 5, *CaNAC*50 and *CaNAC*56 were not expressed in the leaves in the treatment groups.

Under SA treatment, the expression of five genes (*CaNAC*23, *CaNAC*29, *CaNAC*35, *CaNAC*72, and *CaNAC*79) was upregulated and two genes (*CaNAC*60 and *CaNAC*102) were downregulated. The expression of six other genes (*CaNAC*13, *CaNAC*20, *CaNAC*27, *CaNAC*37, *CaNAC*75, and *CaNAC*92) were upregulated at first, and then downregulated. The expression value of twelve genes (*CaNAC*13, *CaNAC*20, *CaNAC*27, *CaNAC*33, *CaNAC*35, *CaNAC*37, *CaNAC*41, *CaNAC*72, *CaNAC*75, *CaNAC*79, *CaNAC*86, and *CaNAC*92) peaked after 3 h of treatment. In addition, *CaNAC*20, *CaNAC*35, *CaNAC*41, *CaNAC*72, *CaNAC*75, and *CaNAC*86 were induced by more than 3-fold after 3 h of SA treatment. As shown in Figure 5, *CaNAC*50 and *CaNAC*56 were also not expressed in the leaves in the treatment groups.

Under MeJA treatment, the expression of seven genes (*CaNAC*20, *CaNAC*35, *CaNAC*37, *CaNAC*61, *CaNAC*72, *CaNAC*79, and *CaNAC*92) was upregulated and two genes (*CaNAC*29 and *CaNAC*60) were downregulated. The expression value of four genes (*CaNAC*35, *CaNAC*61, *CaNAC*72, and *CaNAC*79) and three genes (*CaNAC*20, *CaNAC*37, and *CaNAC*92) peaked after 6 and 12 h of treatment, respectively. Following 12 h of MeJA treatment, we observed significant upregulation of *CaNAC20* (65-fold) and *CaNAC92* (180-fold) expression. In addition, the expression of five other genes (*CaNAC*13, *CaNAC*23, *CaNAC*33, *CaNAC*53, and *CaNAC*55) were down–up–down–up-regulated after treatment. As shown in Figure 5, *CaNAC*50 and *CaNAC*56 were also not expressed in the leaves after treatment.

### 2.6. Analysis of Stress-Related Cis-Elements in the CaNAC Promoters

**Figure 6:**
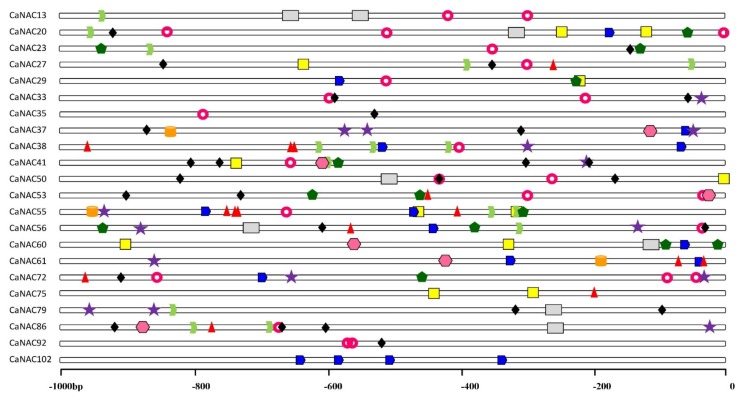
Predicted *cis*-elements in the promoter regions of *CaNAC* genes. The promoter sequences (−1000 bp) of 22 *CaNAC* genes were analyzed. The names of the *CaNAC* genes are shown on the left side of the figure. The number at the bottom indicates the number of the nucleotides to the translation initiation codon, ATG. The elements are identified as follows: green dovetail for the CGTCA element, red triangle for the ABRE element, yellow square for the W-box element, purple five-pointed star for the TCA element, black diamond for the TC-rich repeats element, dark green pentagon for the MBS element, orange oval for the WUN element, blue pentagon for the GARE element, pink circular crown for the HSEs element, the grey rectangle for the ERE element, the rose red hexagon for the LTR element.

We scanned the *cis*-elements involved in the activation of defense-related genes in the promoter regions of *CaNAC* to better understand potential regulatory mechanisms of *CaNAC* genes in response to abiotic or biotic stress responses. The promoter regions (−1000 bp upstream of the translation start site) of all the *CaNAC* genes to which gene expression analysis was applied were examined. The predicted *cis*-elements in the promoter regions of twenty-two *CaNAC* genes are shown in Figure 6.

The ABRE element was found in nine selected promoter regions of *CaNAC*27, *CaNAC*38, *CaNAC*53, *CaNAC*55, *CaNAC*56, *CaNAC*61, *CaNAC*72, *CaNAC*75, and *CaNAC*86. In addition, three ABRE elements were identified in *CaNAC*38, and four were found in *CaNAC*55. The CGTCA element was found in 10 of the selected promoter regions of *CaNAC*13, *CaNAC*20, *CaNAC*23, *CaNAC*27, *CaNAC*38, *CaNAC*41, *CaNAC*55, *CaNAC*56, *CaNAC*79, and *CaNAC*86, and at least two CGTAC elements were found in the *CaNAC*27, *CaNAC*38, *CaNAC*55, and *CaNAC*86 genes. The TCA element was found in 10 selected promoter regions of *CaNAC*33, *CaNAC*37, *CaNAC*38, *CaNAC*41, *CaNAC*55, *CaNAC*56, *CaNAC*61, *CaNAC*72, *CaNAC*79, and *CaNAC*86. In addition, the GARE element was found in 10 selected regions of *CaNAC*20, *CaNAC*29, *CaNAC*37, *CaNAC*38, *CaNAC*55, *CaNAC*56, *CaNAC*60, *CaNAC*61, *CaNAC*72, and *CaNAC*102. It is well known that ABRE, CGTCA, TCA, and GARE *cis*-acting elements are involved in hormone responsiveness. We consistently found these four elements in *CaNAC*38, *CaNAC*55, and *CaNAC*56, thus we suspect that these three *NAC* genes may play a key role in regulating responsiveness to hormones.

As shown in Figure 6, HSE, TC-rich, MBS, and LTR elements were found in sixteen, fourteen, nine, and five selected promoter regions of *CaNAC* genes, respectively. The promoter region of *CaNAC*20 contains three HSE elements, and that of *CaNAC*41 contains four TC-rich elements. These results suggest that *CaNAC*20 and *CaNAC*41 may play a key role in regulating responsiveness to biotic stress. In addition, other stress-related *cis*-elements were detected, including a W-box element in eight genes (*CaNAC*20, *CaNAC*27, *CaNAC*29, *CaNAC*41, *CaNAC*50, *CaNAC*55, *CaNAC*60, and *CaNAC*75), a WUN element in three genes (*CaNAC*37, *CaNAC*55, and *CaNAC*61), and an ERE element in seven genes (*CaNAC*13, *CaNAC*20, *CaNAC*50, *CaNAC*56, *CaNAC*60, *CaNAC*79, and *CaNAC*86).

### 2.7. The Interaction Network of CaNAC Genes

**Figure 7:**
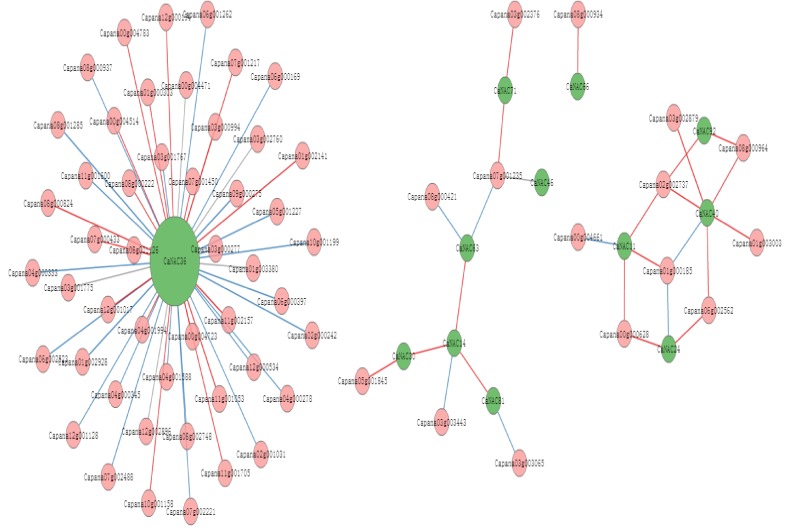
The interaction network of *CaNAC* genes in pepper according to the orthologs in *Arabidopsis***.** The green circles represent the *CaNAC* genes, and the pink circles represent the pepper genes interacting with *CaNAC* genes. The red lines correspond to PCC > 0 (Pearson Correlation Coefficient), the blue lines mean PCC < 0, and the gray lines indicate that the PCC is unknown.

An interaction network of *CaNAC* genes was constructed by making a comparison to orthologs of *Arabidopsis*, to better understand the relationships among the *CaNAC* gene family members. As shown in Table 1, 80.8% (84/104) of *CaNAC* genes were orthologous to 41.9% (49/117) of *Arabidopsis NAC* genes, indicating diversity in the sequences of *NAC* genes in *Arabidopsis* and pepper and that the sequences of *CaNAC* genes were tightly conserved. Positive, negative, and unknown correlations were marked by red, blue, and gray lines in Figure 7, which represent Pearson correlation coefficients of >0, <0, and no correlation, respectively. As shown in Figure 7, a complex functional relationship was observed in the interaction network of *CaNAC* genes; 35 pairs of interacting genes showed positive correlation, 30 showed negative correlation, and 6 were unknown. This suggests that *CaNAC*36 plays a key role in the interaction network, as it fully interacted with 48 genes.

*CaNAC*36 interacted with the Ras-related protein *RAB1A* gene (*Capana*07g000433), *OsRAN*2, a gene orthologous to *Capana*07g000433 in rice, which enhances cold tolerance by promoting the export of intranuclear tubulin and maintaining cell division under cold stress [28]. *CaNAC*36 also interacted with the *SNARE* protein gene (*Capana*12g000194), *GsVAMP*72, a gene orthologous to *Capana*12g000194 in wild soybeans, which is involved in regulating plant salt tolerance and ABA sensitivity [29]. *CaNAC*14 directly interacted with three *CaNAC* genes (*CaNAC*81, *CaNAC*30, and *CaNAC*63), and *CaNAC*40 interacted with mitogen-activated protein kinase genes (*Capana*02g002737, *Capana*06g002562, and *Capana*08g000964), which play important roles in plant growth, development, and in response to a variety of biotic and abiotic stresses [30]. No other direct interactions among *CaNAC* genes were observed.

## 3. Discussion

### 3.1. NAC Gene Expansion and Evolution in Pepper

It has been demonstrated that NAC transcription factors play in important role in regulating stress tolerance against various abiotic and biotic challenges [9]. As a result of the availability of whole genome sequences in recent years, more and more *NAC* gene families have been identified from many different species of plants, such as *Arabidopsis*, rice, apple, and potato. However, very little is known about the NAC family in pepper. In a recently published report, a total of 45 putative NAC sequences were identified from the Pepper Genome Database (PGD, http://peppersequence. genomics.cn) [23]. In the present study, we further revealed an expanded *NAC* gene family in pepper, with a total of 104 members in the pepper genome. However, the *NAC* gene family in pepper is by far the smallest compared to those estimated for other plant species, which include 117 members in *Arabidopsis* [3], 151 in rice [6], and 110 in potato [10]. In particular, the genome size of pepper (3.13 Gb) is much larger than that of *Arabidopsis* (genome size 125 Mb), rice (480 Mb), and potato (840 Mb). By studying subgroups of *NAC* genes in *Arabidopsis* and rice, we found that the number of common subgroup *CaNAC* genes in pepper was much lower than expected as compared to *Arabidopsis* and rice, especially in subgroups 2, 8, 12, and 14. The number of the identified *CaNAC* genes in subgroups 2, 8, 12, and 14 were 3, 5, 6, and 8 in pepper, respectively. However, they are 6, 10, 13, and 13 in *Arabidopsis*, and 7, 8, 6, and 11 in rice, respectively. In our studies, we also identified two new subgroups, subgroup15 and subgroup16, which could not be further divided into subgroups in Group I. A similar phenomenon has been observed in other plants such as potato and is possibly related to the specificity of each species [10]. Further, we also found that 23 *NAC* genes belong to the *Solanaceae* specific subfamily (Figure 2). The number of *NAC* genes is lower in pepper as compared to other *Solanaceae* specific subfamilies such as tobacco (50 *NAC* genes) and potato (36 *NAC* genes). Our results provide further evidence that there is one NAC subfamily which is exclusive to the *Solanaceae* family.

It is well known that gene duplication events are important in the rapid expansion and evolution of gene families [25]. Tandem gene duplication of NAC transcription factors has been observed in many plant species, such as *Arabidopsis*, rice, *Populus*, potato, etc. In potato, one of the most important *Solanaceae* family plants, 24.5% of *StNAC* (27/110) were found to be tandemly duplicated [10]. In our investigation, 30.8% (32/104) of *CaNAC* genes were found to evolve from tandem gene duplication (Figure 1). Therefore, tandem gene duplication plays an important role in *NAC* gene family expansion in pepper. However, in comparing the number of *NAC* genes with the size of the genome, the reason for the low number of *NAC* genes might be due to whole-genome duplication (WGD) events in pepper [25,26].

### 3.2. CaNAC Proteins Play Important Roles in Various Biological Processes

Most of the NAC proteins that have been studied to date are involved in the response to abiotic and biotic stress and in the regulation of developmental processes. However, very few NAC transcription factors involved in the biotic stress response have been identified in pepper. Therefore, the goal of this study was to obtain more insight into the expression patterns and putative functions of *CaNAC* genes in response to biotic or abiotic stresses. The expression levels of 22 *CaNAC* genes under seven stress treatments (salt, heat shock, drought, *Phytophthora capsici*, ABA, SA, and MeJA) were calculated (Figure 5). Almost all 22 *CaNAC* genes were induced by these treatments, except *CaNAC*20 under salt and drought treatment, *CaNAC*50 and *CaNAC*56 under heat shock and hormone treatment, and *CaNAC*56 under *Phytophthora capsici* infection. These results indicate that the accumulation of *CaNAC* effectively reduces the damage from biotic or abiotic stress.

In the present study, the expression of the *CaNAC*72 gene was upregulated under stress treatment, except for *Phytophthora capsici* infection. Under salt and drought treatments (Figure 5), *CaNAC*72 gene showed relatively higher intensities, increasing more than 600- and 70-fold, respectively. We also found that *CaNAC*92 was significantly upregulated by salt (60-fold increase) and drought treatment (18-fold increase). As shown in Table 1, two *Arabidopsis NAC* genes, AT3G15500 (*ANAC*055) and AT1G69490 (*ANAC*029), were orthologous genes to *CaNAC*72 and *CaNAC*92, respectively. According to the phylogenetic tree analysis, *CaNAC72* and *CaNAC*92 were attributed to subgroups 1(5) and 1(4), which shared high identity with subgroups *AtNAC*3 and *AtNAP* of *Arabidopsis*, respectively. In a previous study, Kim (2014) reported that the expression of *AtNAP* and *ANAC*055 increased in a leaf during aging, and that these two genes were senescence-associated NAC transcription factors, as candidate downstream components of ETHYLENE-INSENSITIVE2 (EIN2) [31]. We suspect that *NAC* genes from subgroups *AtNAC*3 and *AtNAP* may play important roles not only in response to abiotic stress, but also in plant development. However, this theory requires further investigation to be confirmed.

In tomato, the expression of *SISRN*1 was significantly induced by infection with *B. cinerea* or *P. syringae* pv. *tomato* DC3000, leading to a 6- to 8-fold higher expression than in the mock-inoculated plants [19]. *SISRN*1 has high identity with *CaNAC*23, and *CaNAC*23 also exhibited an increased expression pattern under *P. capsici* infection stress in this study. These results suggest that *CaNAC2*3 can be induced by pathogens. Further, we also found that the expression of *CaNAC*23 was significantly induced by salt, drought, and heat shock treatment. Thus, *CaNAC*23 genes may play important roles in response to abiotic or biotic stress. Similarly, the expression of *StNAC*030 was significantly induced by salt, heat, and ABA [10] in potato, and *CaNAC*27, the gene orthologous to *StNAC*030, was also significantly induced by salt, heat, and ABA. *CaNAC27* was also induced by drought and MeJA in the present study, suggesting that *CaNAC*27 genes may play important roles in response to abiotic or biotic stress. Thus, we suspect that orthologous NAC genes in the *Solanaceae* family may have similar gene functions; however, their functional characterization would be required to ascertain if they play similar roles in plant processes.

It was reported that two maize NAC transcription factors, namely, *ZmNAC*41 and *ZmNAC*100, are induced in leaves infected with *Colletotrichum graminicola* [32]. However, sequence information about these ZmNAC transcription factors is unavailable, therefore we could not identify orthologous genes in the *CaNAC* family. These results imply that these NAC members might be regulators of responses to various biotic stresses. To date, there are only three *CaNAC* genes with expression models that have been characterized after biotic or abiotic inoculation treatment, while the biological and cellular functions of most *CaNAC* genes remain largely unknown [20,21,22]. The current investigation identified several *CaNAC* genes that might be involved in stress defense and provides clues for the selection of candidate genes for further studies.

## 4. Materials and Methods

### 4.1. Sequence Database Searches

*Arabidopsis* NAC protein sequences were downloaded from TAIR (Available online: ftp://ftp.arabidopsis.org) [33]. Rice NAC protein sequences were obtained from rice full-length cDNA data (KOME) [34]. The pepper annotated genome sequences were obtained from the Pepper Genome Database (PGP, available online: http://peppergenome.snu.ac.kr and PGD, available online: http://peppersequence. genomics.cn) [25,26]. In a previous study, we obtained 44.6 Gb databases from RNA-Seq using resistant and sensitive gene pools against *P. capsici* in pepper and identified 47 NAC transcription factors after gene annotations [35]. In the current study, 47 pepper NAC proteins were used as query sequences for searching candidate sequences (*e* value was ≤e^−10^) against the two pepper genome databases using the BLAST tool (https://solgenomics.net/tools/blast/. The candidate sequences were confirmed for the presence of NAC domains using the Hmmsearch program (HMMER 3.1, available online: http://hmmer.janelia.org/). The putative NAC sequences, confirmed by Hmmsearch in the two pepper genome databases, were in turn used to search the pepper predicted proteins until no new sequences were found. The NAC sequences in two different pepper genome databases were then blasted using DNAMAN software (8.0, Lynnon Biosoft, San Ramon, CA, USA).

### 4.2. Multiple Sequence Alignment, Gene Chromosomal Location, and Phylogenetics Analysis

Approximately 160 amino acid sequences spanning the NAC core domain of all *CaNAC* proteins, 20 selected *Arabidopsis* NAC proteins and rice NAC proteins belonging to different group/subgroups [3], and 9 tobacco NAC proteins belonging to the *Solanaceae* specific NAC subfamily, were used to create multiple protein sequence alignments using Clustal X 2.1 (University College Dublin, Belfield, Dublin, Ireland) with default settings [36]. The gene chromosomal locations were obtained by the pepper gene annotation giff3 file, downloaded from the Pepper Genome Databases (Available online: http://peppersequence.genomics.cn). The NAC domain boundary was defined following a previously described method [3]. The neighbor-joining method was used to construct the phylogenetic tree, based on the amino acid sequence of NAC domains using MEGA 6.06 [37]. The parameters used in tree construction were the Jones-Thornton-Taylor (JTT) model plus gamma-distributed rates, as determined by ProTest 3.0 and 1000 bootstraps [38]. The tandemly and segmentally duplicated genes were defined as an array of two or more homologous genes within a 100-kb range distance, or low-copy repeats of DNA segments (blocks of sequence ≥1 kb in length and showing ≥90% sequence identity), respectively [39].

### 4.3. Motif Composition Analysis of CaNAC Proteins

The MEME 4.12.0 online program (Available online: http://meme-suite.org/) was used for the identification of motifs in the *C. annuum* NAC protein sequences. The optimized parameters of MEME were employed as follows: number of repetitions, any; maximum number of motifs, 20; optimum width of each motif, between 6 and 50 residues [40].

### 4.4. Treatments of Pepper Plants with Various Biotic and Abiotic Stresses

Accession PI201234, which is highly resistant to *P. capsici*, was used throughout this study [41]. The seedlings were grown in a growth chamber for 26 °C (16 h/day) and 20 °C (8 h/night) till reaching the age of six true leaves and were then treated with heat shock (42 °C), salt (300 Mm NaCl), drought (400 Mm mannitol), *P. capsici* (10^5^ spores mL^−1^), salicylic acid (SA, 100 μM), methyl jasmonate (MeJA, 100 μM), and abscisic acid (ABA, 100 μM) for 24 h. The samples were prepared and collected using the method reported in our previous study [41]. The roots treated with drought or *P. capsici* spore suspension inoculation, along with the leaves treated with salt, heat shock, SA, MeJA, or ABA were also collected separately at 0, 3, 6, 12, and 24 h for RNA isolation [42].

### 4.5. Real-Time Quantitative RT-PCR

Total RNA extraction, cDNA synthesis and real-time quantitative PCR (qRT-PCR) analysis were carried out using the methods reported in our previous study [43]. A total of 22 *CaNAC*s belonging to the different subgroups were randomly selected for gene expression analysis under abiotic or biotic stresses, and the data of three biological replicates were analyzed. *Actin1* (NCBI accession number GQ339766) was used as the internal reference gene.

### 4.6. Search for Cis-Acting Elements in the Promoters of CaNAC Genes

Upstream regions (−1000 bp) of the *CaNAC* genes which were selected for gene expression analysis, were derived from Sol Genomics Network (Available online: https://solgenomics.net/) and were searched for regulatory elements, including CGTCA (*cis*-acting regulatory element involved in the MeJA-responsiveness), ABRE (*cis*-acting element involved in the abscisic acid responsiveness), W-box (binding site for the WRKY transcription factor in the defense response), TCA (*cis*-acting element involved in salicylic acid responsiveness), TC-rich repeats (*cis*-acting element involved in the defense and stress responsiveness), MBS (MYB binding site involved in drought induction), WUN (wound-responsive element), GARE (gibberellins-responsive element), HSE (*cis*-acting element involved in heat stress responsiveness), ERE (ethylene-responsive element), and LTR (low temperature-responsive element), in the promoters using the PlantCARE (Available online: http://bioinformatics.psb.ugent.be/webtools/plantcare/html/) and PLACE databases [44,45].

### 4.7. Prediction of CaNACs Protein–Protein Interaction Network

An interaction network of *CaNAC* proteins was constructed to understand the relationships among *CaNAC* gene family members and other genes. As described in detail previously [46], the interolog from *Arabidopsis* was used for predicting protein–protein interaction networks of *CaNAC* genes. An interaction network of *Arabidopsis* NAC proteins was constructed, the *Arabidopsis* NAC proteins were mapped to *CaNAC* proteins on the basis of their homologous relationships, and an interaction network of *CaNAC* proteins was drawn by Cytoscape.

## 5. Conclusions

A genome-wide analysis of the *CaNAC* gene family in pepper was performed to reveal gene location, gene structure, gene phylogeny, conserved motifs, stress-related *cis*-element, gene expression response to seven different abiotic or biotic stresses, and interaction network of *CaNAC*s proteins*.* The vast majority of the 22 *CaNAC* genes showed a response to different stress types, displaying distinct expression patterns. In addition, one NAC subfamily that is exclusive to the *Solanaceae* family was identified. 

## Figures and Tables

**Table 1:** NAC transcript factor family in pepper.

Gene	Annotation ID	Chr	Group	Introns	PI	AA	WT	At Orthologous Gene	*e*-Value
*CaNAC*1	*Capana*00g000175/*Ca*10g20640	chr 10	II(26)	1	4.21	569	64.52	AT2G43000	0.35
*CaNAC*2	*Capana*00g001681/*Ca*10g20530	chr 10	II(26)	0	8.47	100	11.80	AT2G43000	0.097
*CaNAC*3	*Capana*00g001918/*Ca*04g14740	chr 04	I(8)	1	4.56	586	65.85	AT1G34190	1 × 10^−13^
*CaNAC*4	*Capana*00g003238/*Ca*07g03900	chr 07	-	0	7.45	137	16.09	-	-
*CaNAC*5	*Capana*00g004670/*Ca*06g17870	chr 06	I(14)	2	6.42	317	36.12	AT3G15170	4 × 10^−21^
*CaNAC*6	*Capana*00g004843/*Ca*06g14960	chr 06	II(25)	0	4.88	326	37.03	AT5G04400	3.3
*CaNAC*7	*Capana*00g004888/*Ca*06g15350	chr 06	II(23)	0	4.36	534	60.80	-	-
*CaNAC*8	*Capana*01g000033	chr 01	I(12)	2	6.79	420	48.06	AT1G32770	1 × 10^−18^
*CaNAC*9	*Capana*01g000034	chr 01	I(12)	2	6.79	420	48.06	AT1G32770	1 × 10^−18^
*CaNAC*10	*Capana*01g000515/*Ca*01g04740	chr 01	I(15)	2	7.34	336	38.87	AT1G62700	2 × 10^−47^
*CaNAC*11	*Capana*01g000650/*Ca*06g18770	chr 01	I(14)	3	7.57	350	38.97	AT5G39610	5 × 10^−30^
*CaNAC*12	*Capana*01g001228/*Ca*01g09650	chr 01	I(14)	2	7.70	352	39.20	AT5G18270	7 × 10^−15^
*CaNAC*13	*Capana*01g001521/*Ca*01g11960	chr 01	II(25)	0	5.36	276	31.72	AT1G60340	2.7
*CaNAC*14	*Capana*01g002000/*Ca*00g54690	chr 01	I(3)	2	10.12	248	28.34	AT5G13180	1 × 10^−8^
*CaNAC*15	*Capana*01g002406/*Ca*01g17160	chr 01	I(12)	1	8.07	303	34.94	AT4G10350	6 × 10^−57^
*CaNAC*16	*Capana*01g003266/*Ca*01g26660	chr 01	II(26)	0	4.08	461	52.28	AT2G43000	0.29
*CaNAC*17	*Capana*01g004120/*Ca*01g30550	chr 01	I(14)	2	9.15	353	39.92	AT2G24430	1 × 10^−21^
*CaNAC*18	*Capana*01g004489/*Ca*01g34750	chr 01	I(12)	1	6.57	313	35.95	AT1G32770	3 × 10^−25^
*CaNAC*19	*Capana*02g000057/*Ca*02g06450	chr 02	I(9)	5	4.92	577	66.03	AT3G03200	2 × 10^−9^
*CaNAC*20	*Capana*02g000302/*Ca*02g03060	chr 02	I(2)	1	6.52	252	29.08	AT1G62700	4 × 10^−5^
*CaNAC*21	*Capana*02g001393/*Ca*02g04820	chr 02	I(16)	2	6.26	322	36.74	AT1G34190	6 × 10^−11^
*CaNAC*22	*Capana*02g002153/*Ca*02g17910	chr 02	II(25)	0	8.56	246	28.72	AT5G39690	8.5
*CaNAC*23	*Capana*02g002277/*Ca*02g20290	chr 02	I(9)	5	6.27	632	72.58	AT3G03200	1 × 10^−16^
*CaNAC*24	*Capana*02g002611/*Ca*00g89790	chr 02	I(15)	0	5.17	348	39.97	AT2G18060	6 × 10^−85^
*CaNAC*25	*Capana*02g002612/*Ca*00g89790	chr 02	I(15)	1	5.17	348	39.97	AT2G18060	1 × 10^−75^
*CaNAC*26	*Capana*02g002682/*Ca*02g22730	chr 02	II(23)	0	7.09	296	33.97	-	-
*CaNAC*27	*Capana*02g003352/*Ca*02g28070	chr 02	I(14)	2	7.74	326	37.31	AT5G07680	3 × 10^−37^
*CaNAC*28	*Capana*02g003374/*Ca*02g28290	chr 02	I(16)	1	6.52	397	44.85	AT5G39820	2 × 10^−11^
*CaNAC*29	*Capana*02g003557/*Ca*02g30090	chr 02	II(19)	5	4.81	387	43.11	AT5G18270	2 × 10^−4^
*CaNAC*30	*Capana*03g000802/*Ca*12g13470	chr 03	I(14)	2	7.53	332	38.10	AT3G29035	3 × 10^−34^
*CaNAC*31	*Capana*03g000991/*Ca*03g28100	chr 03	-	0	4.38	236	26.39	-	-
*CaNAC*32	*Capana*03g001014/*Ca*03g27130	chr 03	II(23)	0	4.94	245	27.96	-	-
*CaNAC*33	*Capana*03g001027/*Ca*03g27300	chr 03	II(23)	0	4.67	284	32.05	-	-
*CaNAC*34	*Capana*03g001525/*Ca*03g23090	chr 03	I(9)	1	4.77	248	28.75	AT3G17730	9 × 10^−34^
*CaNAC*35	*Capana*03g001657/*Ca*03g22350	chr 03	I(3)	2	6.64	237	27.06	AT4G17980	4 × 10^-5^
*CaNAC*36	*Capana*03g001780/*Ca*03g21000	chr 03	I(10)	5	4.65	632	69.87	AT4G35580	0.002
*CaNAC*37	*Capang*03g002541/*Ca*03g15410	chr 03	I(15)	1	9.14	170	19.75	AT1G12260	5 × 10^−19^
*CaNAC*38	*Capana*03g002678/*Ca*02g15520	chr 03	II(20)	2	8.46	290	33.07	AT1G28470	8 × 10^−7^
*CaNAC*39	*Capana*03g003003/*Ca*03g14470	chr 03	I(12)	2	7.24	326	37.00	AT1G79580	3 × 10^−19^
*CaNAC*40	*Capang*03g003315/*Ca*00g46270	chr 03	I(13)	2	6.24	290	33.35	AT4G28530	3 × 10^−6^
*CaNAC*41	*Capana*04g000051/*Ca*04g23340	chr 04	-	2	6.71	390	44.12	AT5G18270	5 × 10^−21^
*CaNAC*42	*Capana*04g000414	chr 04	II(18)	4	4.67	350	38.80	-	-
*CaNAC*43	*Capana*04g000417/*Ca*04g20070	chr 04	II(18)	4	4.42	371	40.96	-	-
*CaNAC*44	*Capana*04g000587	chr 04	I(13)	2	6.04	279	31.13	-	-
*CaNAC*45	*Capana*04g001537/*Ca*00g03050	chr 04	I(6)	2	8.33	290	33.39	AT1G01720	9 × 10^−16^
*CaNAC*46	*Capana*05g000569/*Ca*05g04410	chr 05	I(6)	2	7.45	306	34.95	AT5G08790	1 × 10^−39^
*CaNAC*47	*Capana*05g001291	chr 05	-	0	4.26	140	16.32	-	-
*CaNAC*48	*Capana*05g001292	chr 05	-	2	6.16	459	51.28	-	-
*CaNAC*49	*Capana*05g001365/*Ca*05g09500	chr 05	II(20)	2	6.94	486	53.97	AT4G29230	3 × 10^−47^
*CaNAC*50	*Capana*05g001593/*Ca*11g04580	chr 05	I(16)	2	7.13	410	46.22	AT1G26870	3 × 10^−25^
*CaNAC*51	*Capana*05g002476/*Ca*00g86410	chr 05	I(8)	5	4.44	551	61.62	AT3G10500	3 × 10^−8^
*CaNAC*52	*Capana*05g002477/*Ca*00g86400	chr 05	I(8)	3	5.26	396	44.64	AT5G07680	4 × 10^-12^
*CaNAC*53	*Capana*06g000136/*Ca*06g27940	chr 06	I(13)	2	6.14	287	33.46	AT4G28530	2 × 10^−10^
*CaNAC*54	*Capana*06g000341/*Ca*06g25420	chr 06	II(26)	0	4.40	467	53.68	AT1G34180	0.073
*CaNAC*55	*Capana*06g000625/*Ca*06g22780	chr 06	I(11)	3	9.14	408	45.91	AT3G44290	3 × 10^−16^
*CaNAC*56	*Capana*06g000752/*Ca*06g21590	chr 06	I(10)	5	4.56	637	70.12	AT3G10490	0.006
*CaNAC*57	*Capana*06g001075/*Ca*06g17290	chr 06	II(17)	2	5.08	379	42.94	AT3G15170	0.93
*CaNAC*58	*Capana*06g001093	chr 06	II(17)	1	4.54	133	15.37	AT3G15170	0.08
*CaNAC*59	*Capana*06g001387/*Ca*06g14050	chr 06	I(15)	2	6.24	121	37.60	AT4G36160	1 × 10^−18^
*CaNAC*60	*Capang*06g001560/*Ca*06g13110	chr 06	I(9)	3	5.45	342	38.48	AT1G32510	2 × 10^−10^
*CaNAC*61	*Capana*06g001739/*Ca*06g11310	chr 06	I(6)	2	6.72	294	33.67	AT5G08790	3 × 10^−12^
*CaNAC*62	*Capana*06g002411/*Ca*06g06410	chr 06	I(15)	2	6.63	347	40.20	AT1G12260	8 × 10^−29^
*CaNAC*63	*Capana*06g002441/*Ca*00g77790	chr 06	I(9)	3	5.24	334	37.91	AT5G63790	1 × 10^−5^
*CaNAC*64	*Capana*06g002936	chr 06	-	1	9.83	136	15.88	AT3G10490	0.005
*CaNAC*65	*Capana*06g003012/*Ca*06g01680	chr 06	I(13)	2	6.24	290	33.39	AT4G28530	3 × 10^−12^
*CaNAC*66	*Capana*07g000058/*Ca*07g00500	chr 07	II(17)	2	5.42	415	47.22	AT3G15500	4 × 10^−9^
*CaNAC*67	*Capana*07g000215/*Ca*07g02120	chr 07	II(22)	0	4.42	196	22.85	AT1G34180	5 × 10^−4^
*CaNAC*68	*Capana*07g001015	chr 07	-	1	10.03	238	26.98	AT5G39820	1 × 10^−8^
*CaNAC*69	*Capana*07g001769/*Ca*00g84200	chr 07	II(20)	3	7.65	325	36.58	AT4G28500	1 × 10^−21^
*CaNAC*70	*Capana*07g002072/*Ca*07g16790	chr 07	II(25)	0	9.70	235	27.48	AT1G34190	1.5
*CaNAC*71	*Capana*07g002159/*Ca*07g17460	chr 07	I(14)	1	8.85	323	36.23	AT5G53950	5 × 10^−70^
*CaNAC*72	*Capana*07g002219/*Ca*07g18020	chr 07	I(5)	2	7.44	338	37.67	AT3G15500	5 × 10^−27^
*CaNAC*73	*Capana*07g002220/*Ca*07g02180	chr 07	I(7)	8	8.70	340	38.00	-	-
*CaNAC*74	*Capana*07g002471/*Ca*07g21320	chr 07	I(16)	2	7.54	323	37.20	AT2G43000	3 × 10^−9^
*CaNAC*75	*Capana*07g002485/*Ca*07g21470	chr 07	I(13)	2	8.61	136	15.53	AT1G56010	3 × 10^−18^
*CaNAC*76	*Capana*08g000884/*Ca*08g06780	chr 08	II(24)	0	7.30	232	26.86	-	-
*CaNAC*77	*Capana*08g000885/*Ca*08g06790	chr 08	II(24)	0	6.79	178	20.64	-	-
*CaNAC*78	*Capana*08g001727/*Ca*08g13660	chr 08	I(2)	1	7.16	408	46.32	AT2G02450	3 × 10^−75^
*CaNAC*79	*Capana*08g001999/*Ca*08g15460	chr 08	II(21)	2	4.48	195	22.63	AT5G64530	5 × 10^−10^
*CaNAC*80	*Capana*09g000105/*Ca*09g18600	chr 09	II(21)	1	4.92	314	36.51	-	-
*CaNAC*81	*Capana*09g000936/*Ca*09g12970	chr 09	I(5)	2	9.45	350	39.62	AT4G27410	9 × 10^−44^
*CaNAC*82	*Capana*09g002022/*Ca*03g21000	chr 09	-	0	10.32	103	12.00	AT4G35580	3 × 10^−4^
*CaNAC*83	*Capana*09g002444/*Ca*09g00160	chr 09	I(1)	1	9.83	161	18.66	AT5G39610	0.002
*CaNAC*84	*Capana*10g000063/*Ca*10g00460	chr 10	I(7)	4	8.41	342	39.05	AT1G61110	2 × 10^−1^
*CaNAC*85	*Capana*10g000683/*Ca*11g08290	chr 10	I(12)	2	6.46	341	39.09	AT2G46770	2 × 10^−51^
*CaNAC*86	*Capana*10g001138/*Ca*10g09910	chr 10	I(2)	1	6.68	299	34.40	AT3G12977	0.047
*CaNAC*87	*Capana*10g002356/*Ca*10g20640	chr 10	II(26)	0	4.17	578	65.18	AT2G43000	0.023
*CaNAC*88	*Capana*10g002363/*Ca*10g20640	chr 10	II(26)	1	5.72	421	48.33	AT2G43000	0.26
*CaNAC*89	*Capana*11g000328	chr 11	I(1)	2	8.56	256	29.67	AT3G44350	7 × 10^−13^
*CaNAC*90	*Capana*11g000346/*Ca*11g16490	chr 11	I(11)	3	8.94	414	46.60	AT3G44290	8 × 10^−14^
*CaNAC*91	*Capana*11g000795/*Ca*11g12260	chr 11	II(17)	2	6.51	344	40.34	AT1G34180	2× 10^−5^
*CaNAC*92	*Capana*11g001813/*Ca*11g04440	chr 11	I(4)	2	7.57	294	33.85	AT1G69490	2 × 10^−26^
*CaNAC*93	*Capana*11g001932/*Ca*10g13700	chr 11	II(24)	0	10.07	167	19.90	-	-
*CaNAC*94	*Capana*11g001935/*Ca*10g13720	chr 11	II(24)	0	6.68	218	25.63	-	-
*CaNAC*95	*Capana*11g002231/*Ca*11g01550	chr 11	II(19)	3	5.12	481	54.73	AT1G25580	1 × 10^−15^
*CaNAC*96	*Capana*12g002058/*Ca*12g07920	chr 12	-	3	4.44	719	79.51	AT1G33280	0.007
*CaNAC*97	*Capana*12g002348/*Ca*12g05890	chr 12	II(22)	0	10.15	154	18.04	-	-
*CaNAC*98	*Capana*12g002352/*Ca*12g05910	chr 12	II(22)	0	4.75	269	31.41	AT5G62380	0.011
*CaNAC*99	*Capana*12g002357/*Ca*12g06210	chr 12	II(22)	0	9.35	224	26.75	AT1G34180	0.14
*CaNAC*100	*Capana*12g002360/*Ca*12g05910	chr 12	II(22)	0	4.63	268	31.17	AT5G62380	0.011
*CaNAC*101	*Capana*12g002456/*Ca*12g04970	chr 12	I(8)	5	4.35	476	52.88	AT3G10500	1 × 10^−9^
*CaNAC*102	*Capana*12g002457/*Ca*12g04950	chr 12	I(8)	3	5.17	426	47.63	AT1G34180	1 × 10^−9^
*CaNAC*103	*Capana*12g002917/*Ca*12g00080	chr 12	II(21)	2	4.27	195	22.09	-	-
*CaNAC*104	*Capana*12g002918/*Ca*12g00080	chr 12	II(21)	2	4.27	195	22.96	-	-

Chr: chromosome; WT: molecular weight (kDa); AA: amino acid; PI: isoelectric point.

## Supplementary Materials

Supplementary materials can be found at http://www.mdpi.com/1422-0067/19/4/1028/s1.
